# Methotrexate-Induced Stroke-Like Syndrome: A Typical Presentation of a Rare Complication

**DOI:** 10.7759/cureus.40004

**Published:** 2023-06-05

**Authors:** Mariana Leitão Santos, Sílvia Silva, Amélia Moreira, Augusto Ribeiro

**Affiliations:** 1 Pediatric Medicine, Hospital de Braga, Braga, PRT; 2 Pediatric Oncology, IPO Porto (Portuguese Oncology Institute of Porto), Porto, PRT; 3 Pediatric Intensive Care Unit, Centro Hospitalar e Universitário de São João, Porto, PRT

**Keywords:** neuroimage, methotrexate neurotoxicity, neurotoxicity, stroke like syndrome, b-cell all (acute lymphoblastic leukemia)

## Abstract

Methotrexate (MTX) is one of the mainstay drugs used in acute lymphoblastic leukemia (ALL) management; however, it can cause damage to the central nervous system (CNS), typically to the subcortical white matter. Stroke-like syndrome is one particular form of MTX-related neurotoxicity that occurs within 21 days of methotrexate administration (intrathecal or high-dose intravenous treatment). The clinical picture comprehends fluctuating neurological symptoms evoking acute cerebral ischemia or hemorrhage (paresis or paralysis, speech disorders - aphasia and/or dysarthria, altered mental status, and occasionally seizures), with spontaneous resolution in the majority of cases, without other identifiable cause. The typical neuroimage includes areas of restricted diffusion on diffusion-weighted imaging and non-enhancing T2 hyper-intense lesions in the white matter, on brain MRI. We report a 12-year-old boy with low-risk B-ALL without CNS involvement, who presented to the emergency department with complaints of sudden paresis of the four limbs (more severe on the right side), aphasia, and confusion. He had received one dose of intrathecal MTX 11 days prior to this episode. An angio-MRI of the brain revealed bilateral restricted diffusion areas in the centrum semiovale, and symptoms fluctuated until complete neurological recovery without any medical intervention, which is very suggestive of MTX-related neurotoxicity. This case illustrates a rare complication of MTX administration that presented with typical clinical and radiological characteristics, in an adolescent with hematological malignancy who experienced swift and full neurological recovery.

## Introduction

Methotrexate (MTX) is an important chemotherapeutic agent used in acute lymphoblastic leukemia (ALL) treatment. It is a folic acid antagonist, and in intravenous high-dose or intrathecal administration, it can be associated with demyelination, white matter necrosis, loss of oligodendroglia, axonal swelling microcystic encephalomalacia, and atrophy in the deep cerebral white matter [1-3]. The typical damage inflicted on the central nervous system (CNS) white matter is termed leukoencephalopathy [3].

Currently, the overall incidence of symptomatic neurotoxicity related to frontline ALL therapy is about 10-12% [4]. Neurotoxicity specifically induced by MTX can account for acute to subacute symptoms or delayed/chronic presentation [5]. The incidence of subacute MTX neurotoxicity is still unclear, diverging in different studies. Some authors report that 3% to 7% of these patients experience symptoms of subacute methotrexate neurotoxicity, which includes stroke-like syndrome (SLS), encephalopathy, speech disturbances, and seizures [4,6]. Still others describe an incidence ranging from 0.8% to 3.8% and in recent papers, it can be as low as 0.2% [5,6].

We report a case of subacute MTX neurotoxicity, which resolved very rapidly without the need for any intervention.

## Case presentation

A 12-year-old Caucasian male presented with unremarkable past medical history except for hospitalization in the neonatal period due to transient tachypnea and early onset sepsis. He had a recent diagnosis of B-cell acute lymphoblastic leukemia (ALL), average low-risk group (AR1), meaning no testicular or CNS involvement, with a good pre-phase response. The diagnosis was made after finding lumbar pathologic fractures (T8-S1). As a consequence of the oncologic disease, he was medicated with alprazolam for anxiety. He was receiving chemotherapy based on the European Organization for Research and Treatment of Cancer- EORTC 58081 protocol, phase IB (which includes periodic triple intrathecal chemotherapy, with methotrexate 12 mg, cytarabine, and corticosteroid).

Three months after the diagnosis, and 11 days after the last intrathecal methotrexate (MTX) following the seventh periodic triple intrathecal chemotherapy, he presented to the emergency department (ER) with sudden generalized muscle weakness and aphasia. He was writing when the episode happened, and the mother recalled that he wasn’t able to do it. He was able to walk to the car, but when he reached the hospital, the deficit was already affecting the four limbs (with deficits more pronounced on the right side). After this episode, he was confused (oriented in time and place, but with difficulties recognizing his mother). There was no associated loss of consciousness, seizure, headache, vision changes, or fever. He had no recent infectious disease or epidemiologic contact.

At the ER, he was apyretic and hemodynamically stable, with oxygen saturation of 100% in room air; his glucose level was 95 mg/dl. He presented a reasonable general appearance with good peripheral perfusion, even if he was noted to be pale. He was hydrated with no visible rash or petechiae. The cardiac and pulmonary auscultation was normal. At the neurological examination, he was alert, orientated to time and space, and cooperative, with slurred speech and isochoric and normally reactive pupils. Eye movements were preserved, without nystagmus. He had right hemiparesis involving the face and right Babinski sign. He had no sensory disorder and scored 8 on the National Institute of Health Stroke Scale (NIHSS). During his time at the ER, he presented fluctuating symptoms.

**Figure 1:**
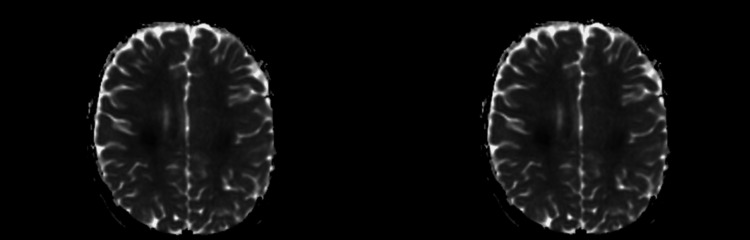
Areas with restriction of diffusion at the centrum semiovale in the ADC map ADC: apparent diffusion coefficient

He went through a blood workup revealing pancytopenia: hemoglobin of 9.6 g/dl, leucocyte count of 1010/uL (with 490 neutrophils), and 60000/uL platelets. He had normal renal function, serum electrolytes, and venous blood gas analysis; fibrinogen was augmented. The contrast head CT findings included an extensive inflammatory sinus disease, involving all paranasal sinuses, with exacerbation at the right maxillary sinus; an anomaly at the venous development in the right frontal topography; and fenestration of the right P1 segment was observed; it was also noted a marked dominance of venous drainage through sigmoid sinus, left transversal, presenting pronounced hypoplasia at the transversal sinus, and right sigmoid (probably constitutional). An angio-MRI of the brain revealed bilateral restricted diffusion areas in the centrum semiovale, evoking methotrexate-related neurotoxicity (Figure 1).

He was admitted to the pediatric intensive care unit (PICU) for neurologic surveillance but he never needed any neuroprotective measures or other supportive interventions (respiratory, hemodynamic, or any other). In fact, since his admission, the deficits resolved very rapidly, and during his stay, he presented a normal physical examination without any complaints. He was transferred to his referral oncologic institution two days later, where he went through an echocardiogram and a carotid artery Doppler ultrasound test, both normal. He restarted the treatment a week behind the protocol. The following intrathecal MTX was given uneventfully, under 24h inpatient surveillance and administration of rescue IV levofolinate disodium 5 mg/m^2^. Presently, he continues in remission of the oncologic disease (normocellular and with normal morphology bone marrow evaluation; negative minimal residual disease; and genetic evaluation), under maintenance treatment.

## Discussion

The mechanism of MTX-induced neurotoxicity is not well-understood but is likely to be multifactorial, including direct damage induced by MTX, and several biochemical alterations that could indirectly disturb the CNS. Accumulation of homocysteine and its metabolites in plasma and cerebrospinal fluid is toxic to vascular endothelium. The release of adenosine with neuronal injury is also a propped mechanism [4].

The MTX SLS definition includes neurotoxicity that occurs in the first 21 days after intravenous or intrathecal MTX, with the typical clinical and neuroimage features, after excluding other causes. Symptoms can last several hours or days, being typically transient and resolving in one to four days. Most patients recover totally, although some patients require rehabilitation. The risk factors include age >10 years, Hispanic ethnicity, elevated serum aspartate transaminase (AST) during induction/consolidation, and coadministration of intrathecal MTX in systemic cytarabine- and cyclophosphamide-containing treatment blocks [4,6]. Our patient combined several risk factors like adolescence and intrathecal MTX.

This case illustrates a rare complication of MTX administration (SLS) that presented with typical clinical and radiological characteristics, and a swift and full neurological recovery, as reported in the literature. This particular form of MTX-related neurotoxicity occurs within 21 days of MTX administration, as a clinical picture of fluctuating neurological symptoms evoking acute cerebral ischemia or hemorrhage (paresis or paralysis, speech disorders - aphasia and/or dysarthria, altered mental status and occasionally seizures), with spontaneous resolution in the majority and no other identifiable cause [4]. The MRI is the preferred exam, and it can reveal the underlying lesions as soon as one hour after the event [3]. The distinctive image is a symmetric hyperintensity at diffusion-weighted imaging, with a reduction of apparent diffusion coefficient (ADC) [7]. These T2 hyperintensities are classically located bilaterally in the periventricular white matter, particularly in the centrum semiovale [3].

As stated, this is typically transient, and most patients can receive subsequent MTX without recurrence of symptoms. Nevertheless, there are reports of very rare cases of poor neurologic outcomes and even fatalities with progressive encephalopathy [8].

Management of MTX-induced SLS is essentially supportive. However, additional folic acid, leucovorin rescue, dexamethasone, aminophylline, and dextromethorphan have been employed in some patients [4].

## Conclusions

This case shows subacute methotrexate neurotoxicity that can mimic a stroke and gathers the typical symptoms and neuroimaging results. The differential diagnosis of neurological disturbances in children with hemato-oncological diseases undergoing chemotherapy is very broad and includes threatening situations like the progression of the disease, stroke, and venous sinus thrombosis. For this reason, it is important that emergency pediatricians recognize stroke-like syndrome, as it allows the early implementation of adequate measures and avoids unnecessary diagnostic or therapeutic procedures. It is important to be aware of this neurological complication of methotrexate whose treatment may reside in clinical observation and supportive care only, as most cases tend to resolve spontaneously.
